# The Genetic Content of Chromosomal Inversions across a Wide Latitudinal Gradient

**DOI:** 10.1371/journal.pone.0051625

**Published:** 2012-12-18

**Authors:** Pedro Simões, Gemma Calabria, João Picão-Osório, Joan Balanyà, Marta Pascual

**Affiliations:** 1 Grup de Biologia Evolutiva/IRBio, Departament de Genètica, Facultat de Biologia, Universitat de Barcelona, Barcelona, Spain; 2 School of Life Sciences, University of Sussex, Brighton, United Kingdom; University of Umeå, Sweden

## Abstract

There is increasing evidence regarding the role of chromosomal inversions in relevant biological processes such as local adaptation and speciation. A classic example of the adaptive role of chromosomal polymorphisms is given by the clines of inversion frequencies in *Drosophila subobscura,* repeatable across continents. Nevertheless, not much is known about the molecular variation associated with these polymorphisms. We characterized the genetic content of *ca*. 600 individuals from nine European populations following a latitudinal gradient by analysing 19 microsatellite loci from two autosomes (J and U) and the sex chromosome (A), taking into account their chromosomal inversions. Our results clearly demonstrate the molecular genetic uniformity within a given chromosomal inversion across a large latitudinal gradient, particularly from Groningen (Netherlands) in the north to Málaga (Spain) in the south, experiencing highly diverse environmental conditions. This low genetic differentiation within the same gene arrangement across the nine European populations is consistent with the local adaptation hypothesis for th evolutionof chromosomal polymorphisms. We also show the effective role of chromosomal inversions in maintaining different genetic pools within these inverted genomic regions even in the presence of high gene flow. Inversions represent thus an important barrier to gene flux and can help maintain specific allelic combinations with positive effects on fitness. Consistent patterns of microsatellite allele-inversion linkage disequilibrium particularly in loci within inversions were also observed. Finally, we identified areas within inversions presenting clinal variation that might be under selection.

## Introduction

Chromosomal inversions are widespread in nature, being present in several animal and plant species. Increasing evidence has been mounting on the role of these mutations in relevant biological processes such as adaptation, speciation and the evolution of sex chromosomes [1],[2]. A well-known example of the adaptive role of chromosomal inversions is given by the chromosomal inversion clines in *Drosophila subobscura*. In this species latitudinal clines for several chromosomal arrangements are well established in Europe [3]–[5] and similar clinal patterns were found both in North and South America few years after colonization demonstrating its adaptive value [6], [7]. Moreover, changes in frequency of these chromosomal arrangements in the three continents are highly correlated with temperature profiles thus tracking climate warming at a worldwide scale ([8], [9] see also [10] for evidence in *D. melanogaster*). These latitudinal clines of chromosomal inversion frequencies are widely maintained in natural populations despite the high dispersal ability and strong gene flow between populations of this species [11].

Several mechanisms have been proposed to explain the maintenance of these polymorphisms in natural populations [1]. Using *Drosophila pseudoobscura* as model, Dobzhansky [12], [13] developed the coadaptation hypothesis based on a selective advantage of inversion heterokaryotypes - individuals heterozygous for chromosomal inversions - due to the existence of positive epistatic interactions between alleles at loci located within gene arrangements of a given population. Since single cross over events within inversion loops give rise to unbalanced gametes, inversion heterokaryotypes present reduced recombination within these regions, preventing the disruption of the interacting sets of alleles and allowing the spread of the inversions through the population [12]. Furthermore, gene exchange between gene arrangements from different populations would disrupt these adaptive complexes and produce less fit allelic combinations. A corollary of this model is the expectation of genetic differentiation between different gene arrangements from the same population and also differentiation within the same gene arrangement across populations [14]. Kirkpatrick and Barton [15] presented another selective hypothesis for the spread of an inversion: as long as chromosomal inversions “harbour” sets of alleles adapted to local conditions, they may be selected even without epistasis. The spread of an inversion under the local adaptation hypothesis is thus explained by the maintenance of a given set of alleles with positive effects on fitness, held together as a result of the reduced recombination in inversion heterokaryotypes.

Moreover, patterns of genetic variation within inverted regions are influenced by the history and age of the inversion and also by the occurrence of gene flow (or flux) in these regions [16]. In some *Drosophila* species high levels of linkage disequilibrium (LD) have been found for genes located within inverted regions, with a LD decrease towards the middle of the inversion ([14], [17] but see [18]). This result is in accordance with the expectation of higher genetic exchange away from breakpoints eventually breaking the initial LD associated with the formation of the inversion. This is because gene conversion rate is expected to be uniformly distributed along the inversion while double crossovers in inversion heterokaryotypes would most likely transfer the central regions of the inversion [19], [20]. The high LD levels found away from breakpoints interspersed with regions of low LD have been taken as evidence of epistasis in loci inside the inversion suggesting that selection is acting on nearby regions, in accordance with the expectations of the coadaptation model [14], [21]. Nevertheless, most studies also report a general absence of genetic differentiation within the same chromosomal arrangement across different *Drosophila* populations [14], [21], [22], a result that contradicts the strict coadaptation model which postulates genetic differentiation of a given gene arrangement between distinct geographical regions. This result can be due to the free gene exchange between same inversion types across populations, magnified by the high dispersal ability in *Drosophila.* Nevertheless, it cannot be excluded that the absence of genetic differences within a given gene arrangement might be due to the fact that such studies were conducted in regions not directly subject to epistatic selection and/or that fitness effects associated with coadaptation are due to several loci each with a small effect (see [14]).

Despite the abovementioned studies, not much is known about possible regions under selection within inverted segments (but see [14], [21], [23]). An important approach that is likely to provide relevant insight on selection targets is the study of molecular genetic clinal variation within chromosomal inversions. In fact, studying genetic variation in populations along environmental gradients can reveal patterns of local adaptation with climate as a candidate selective agent. Linking patterns of genetic differentiation and LD observed across the inversion with specific loci presenting clinal variation might reveal candidate genes and/or regions within inversions subjected to clinal selection (e.g., [21]). Some studies have detected clinal patterns in molecular variants located inside inverted regions. One such example is the *Drosophila melanogaster* cline of *alpha-Gpdh* loci, located inside ln(2L)t, an inversion that also presents a clinal distribution [24]. Kennington *et al.* [21], for instance, found that the markers located within the ln(3R)Payne inversion were those presenting the strongest clinal variation, suggesting selection nearby. On the other hand, McAllister [25] for example did not detect any north–south distribution of genetic variation in chromosome 4 genes of *D. americana* despite clinal variation in gene arrangements.

Caution is needed when interpreting clinal variation as evidence of selection, due to the confounding effects of gene flow. The comparison of patterns obtained in loci located inside *vs.* outside inverted regions might help to differentiate between effects of gene flow *versus* selection on clinal variation [21], [26], [27]. Furthermore, when analyzing clinal variation associated with chromosomal inversions the effect of the inversion itself must be taken into account [24], [28]. The best approach in this case is to study clinal variation of alleles within chromosomes carrying the same gene arrangement.

Despite the well-known latitudinal inversion clines in European *Drosophila subobscura* populations, its underlying clinal variation at the molecular level has not yet been addressed. In this study we characterize the molecular genetic content in three chromosomes (two autosomes and the sex chromosome) associated with the most frequent chromosomal inversions along a wide geographic latitudinal cline. This approach may serve as a tool to detect regions under selection and provide insight into the different hypothesis to explain the maintenance and spread of chromosomal inversions.

Our study involves a total of *ca*. 600 individuals from nine populations of *Drosophila subobscura* ranging up to 24° degrees of latitude in the European continent. These populations were characterized in 19 microsatellite loci mapping inside and outside the most frequent arrangements in the J and U autosomes and the A sex chromosome. More specifically we aim to 1) assess differences in genetic content both within and among chromosomal inversions across a wide geographic gradient, 2) search for conserved Linkage Disequilibrium patterns between molecular markers and inversions, 3) test for molecular genetic clinal variation within inversions and finally 4) compare patterns of linkage disequilibrium and clinal variation to identify genomic regions under selection.

We found clear impact of inversion polymorphisms in the patterns of molecular genetic variation, contributing to the existence of distinct gene pools in inverted regions even in individuals from the same natural location. We also found high levels of genetic differentiation between chromosomal inversions and low differentiation in the same inversion across populations, findings that agree with the local adaptation hypothesis. We also provide evidence on the maintenance of latitudinal clines for inversion frequencies in Western Europe and pinpoint specific regions located within inversions that might be under clinal selection.

## Materials and Methods

### Geographic Samples

**Figure 1:**
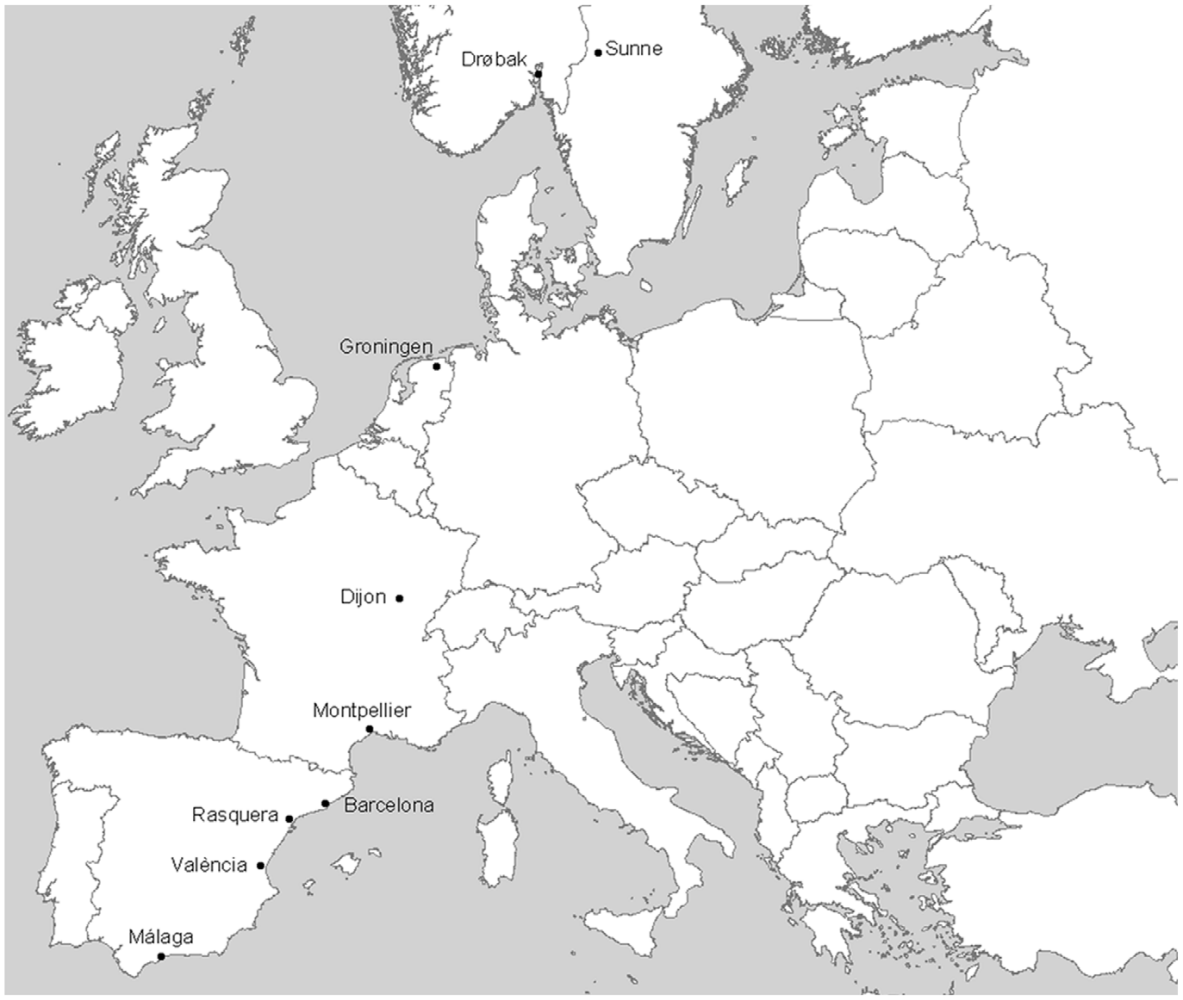
Sampling sites for the *D. subobscura* populations studied.

Wild *Drosophila subobscura* samples were collected from nine European locations (see Figure 1). Collections were performed in the late summer/early fall to reduce seasonality effects on inversion frequency [29] thus allowing more reliable comparisons across years. Individuals from Scandinavian populations of Drøbak (named Dro, situated at 59° 34′N, Norway) and Sunne (Sun, 60° 08′N, Sweden) were collected in August 2005 in a total of 80 and 63 respectively. Samples from Barcelona (Bcn, 41° 25′N, Spain) were obtained in October 2007 with 286 individuals, those from Málaga (Mal, 36° 43′N, Spain), València (Val, 39° 32′N, Spain) and Rasquera (Ras, 40° 57′N, Spain) were collected in October 2008 with a total of 169, 95 and 152 individuals respectively. Individuals from Montpellier (Mon, 43° 35′N, France), Dijon (Dij, 47° 18′N, France) and Groningen (Gro, 53° 13′N, Netherlands) were collected in August/September 2009 in a total number of 221, 344 and 326, respectively (see details in [30]).

### Chromosomal Inversions

The karyotype of *Drosophila subobscura* consists of five acrocentric chromosomes and a dot chromosome [31], corresponding to the ancestral karyotype of the *Drosophila* genus. It is well known that the gene content of these six different elements referred to as Muller’s elements [32] is evolutionary highly conserved, although there is extensive gene reshuffling within elements [33]. This study is focused on three chromosomes: the A chromosome (sex chromosome), the J chromosome (Muller’ D element) and the U chromosome (Muller’s B element).

**Figure 2:**
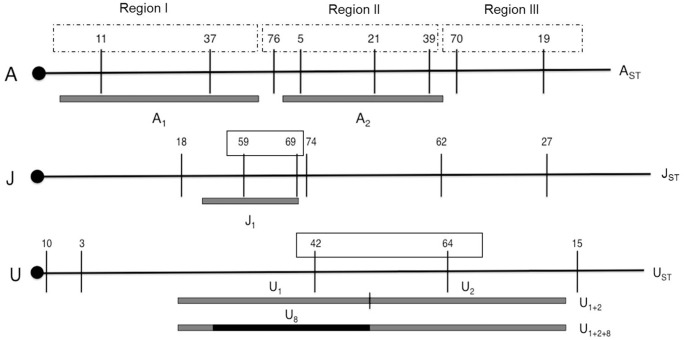
Schematic representation of the microsatellite loci cytological location in chromosomes A, J, and U of *Drosophila subobscura* in relation to the studied arrangements. The centromere on the left is represented by a black circle. Boxes indicate loci located inside inversions (or inside the three regions defined in chromosome A).

In order to score and posteriorly genotype individual chromosomal arrangements from each locality, wild-caught males and males descendant from isofemale lines (F1) were individually crossed with virgin females of the *chcu* strain, an inbred strain with a known homokaryotypic genetic background. One female third-instar larva from each cross was dissected and examined for its polytene chromosomes to obtain information on the arrangements of one set of the chromosomes from the wild. This is possible because the *chcu* strain is homokaryotypic for the chromosomal arrangements A_ST_, J_ST_, U_ST_, E_ST_ and O_3+4_ [34], which allows us to determine for each chromosome one of the arrangements of the wild individual due to the formation of specific inversion loops in the descendants. To obtain chromosome preparations the salivary glands of larvae from crosses with *chcu* strain were stained with 2% orcein in 60% acetic acid mixed 50∶50 with lactic acid. The remains of the larva were preserved in absolute ethanol at −80°C for later DNA extraction. The chromosomal arrangements were classified according to Kunze-Mühl and Müller [35]. The number of individuals assayed per population ranged between 47 individuals in Sunne for the A chromosome and 202 individuals in Barcelona for the J chromosome. The frequency of inversions in each chromosome are reported in Table S1, with the most frequent being: A_ST_, A_1_ and A_2_ for the A chromosome; J_ST_ and J_1_ for the J chromosome; and U_ST_, U_1+2_ and U_1+2+8_ arrangements for the U chromosome (see Figure 2, Table S1).

### DNA Extraction, Microsatellite Amplification and Chromosomal Location

DNAs were individually extracted from the larvae used to score inversion polymorphisms following the protocol described in Pascual et al. [36]. Nineteen previously isolated microsatellites [37] were genotyped in *ca* 600 individuals: dsub11, dsub37, dsub76, dsub05, dsub21, dsub39, dsub70, dsub19 (for the A chromosome); dsub18, dsub59, dsub69, dsub74, dsub62, dsub27 (for the J chromosome); and dsub10, dsub03, dsub42, dsub64, dsub15 (for the U chromosome). These markers were chosen due to their localization on the chromosomes relative to inversions ([33], Figure 2). From each larva of the crosses with *chcu* strain the wild microsatellite allele was easily diagnosed as the *chcu* strain is homozygous (the *chcu* allele had been previously characterized for each microsatellite loci). Analyses were performed taking into account the location of the different loci (inside/outside inverted regions). The definition of loci inside/outside was done by comparison of chromosomes carrying different inversions/arrangements. As such, dsub59 and dsub69 were defined as “loci inside” since their position is inverted in J_1_ chromosomes relative to J_ST_ chromosomes and loci dsub42 and dsub64 were also defined as “inside” since they change position in U_1+2_ chromosomes relative to U_ST_ chromosomes. For the A chromosome, due to the combined effect of non-overlapping inversions which renders difficult the direct comparison explained above, the definition of three regions was used in order to allow a comparison between inverted *vs.* non-inverted chromosomes (Figure 2): region I – with loci dsub11 and dsub37, including the inversion A_1_; region II – with loci dsub76, dsub05, dsub21 and dsub39, including the A_2_ inversion; region III – loci dsub70 and dsub19, no inversion included. A total of 602 individuals were genotyped for the U chromosome, 584 for the J chromosome and 592 for the A chromosome. Sample sizes per inversion and population ranged between 7 and 72 individuals, being around 30 individuals in most cases (Table 1). These sizes do not exactly represent the frequencies of the different inversions in the natural populations although in some cases they were constrained by these frequencies (Table S1).

**Table 1:** Number of analyzed chromosomes (n) and molecular genetic variability (H_e_) for each population and chromosomal arrangement.

Arrangement		MAL	VAL	RAS	BCN	MON	DIJ	GRO	DRO	SUN
**A_ST_**	n	18	19	30	53	30	29	30	50	42
	H_e_	0.869	0.868	0.879	0.894	0.879	0.876	0.892	0.868	0.872
**A_1_**	n	–	–	–	10	14	30	29	16	–
	H_e_	–	–	–	0.842	0.877	0.878	0.873	0.810	–
**A_2_**	n	31	30	28	32	27	24	20	–	–
	H_e_	0.853	0.876	0.871	0.877	0.846	0.875	0.867	–	–
**J_ST_**	n	29	29	28	34	28	30	30	55	43
	H_e_	0.740	0.742	0.803	0.745	0.786	0.784	0.803	0.734	0.697
**J_1_**	n	30	30	30	61	30	30	30	18	17
	H_e_	0.866	0.878	0.882	0.885	0.889	0.877	0.887	0.809	0.816
**U_ST_**	n	–	–	–	7	18	30	30	60	47
	H_e_	–	–	–	0.773	0.814	0.823	0.841	0.823	0.781
**U_1+2_**	n	26	37	30	72	30	30	30	14	–
	H_e_	0.867	0.857	0.826	0.855	0.802	0.833	0.868	0.749	–
**U_1+2+8_**	n	32	27	20	15	9	23	15	–	–
	H_e_	0.818	0.836	0.836	0.849	0.789	0.839	0.836	–	–

Note. (−) represent arrangements not analysed in the population due to their reduced sample size.

The markers were amplified using three different multiplex PCR reactions, one per chromosome, with the Qiagen Multiplex Amplification Kit. The amplification reactions were performed for a total volume of 15 µl with 7.4 µl of Master Mix (Qiagen), 1.5 µl of primer mix (2 µM of each primer) and 1 µl of DNA. Locus dub64 (chromosome U) was amplified individually with the Amersham Taq polymerase. All reactions were performed on an AB GeneAmp PCR System 2700 machine using the following steps: 15 min at 94°C, then 30 cycles of 30 s at 94°C, 30 s at 50°C and 30 s at 72°C; and as final step of 30 min at 60°C. After amplification, the products were visualized in a 2% agarose gel, diluted correspondingly and loaded on an ABI PRISM 3700 automatic sequencer from the Scientific and Technical Services of the University of Barcelona, with CST ROX 70–500 (BioVentures, Inc.) used as internal molecular ladder. Allele sizes were assigned with GeneMapper™ version 3.7 (Applied Biosystems, Inc.).

### Statistical Methods

#### Genetic variability

Levels of genetic variability in microsatellite loci were assessed by measuring the Expected heterozygosity (H_e_, or gene diversity) using the FSTAT software package version 2.9.3.2 [38]. Differences in expected heterozygosity between populations, inversions (or arrangements) and loci located in inverted/non-inverted regions were tested for all chromosomes by Trifactorial ANOVAs. Populations and Arrangements (A_ST_, A_1_ and A_2_; J_ST_ and J_1_; U_1+2_, U_1+2+8_and U_ST_ for each chromosome respectively) were defined as fixed factors and Locus as random factor. Interaction terms between factors were also included in the models. In all analyses, the arcsine of the square root heterozygosity was used as the dependent variable to meet the ANOVA assumptions of normality. ANOVA models were computed with the STATISTICA 8.0 software package.

#### Genetic differentiation within and among chromosomal arrangements

Individuals carrying the same arrangement from a given population were grouped in the same study unit. Molecular genetic differentiation associated with chromosomal arrangements was visualized with a Principal Coordinate Analysis using *F_ST_* pairwise values between and within gene arrangements across populations. *F_ST_* pairwise matrices [39] were obtained from Arlequin v3.5.1.3 [40] since this program allows dealing with haploid microsatellite data. Analyses were also separately performed for microsatellite loci located inside and outside inverted segments. AMOVA locus-by-locus were performed and global *F_ST_* values were obtained for each locus considering either comparisons of all arrangements from the several populations in the analysis (*F_ST_* values between arrangements) or only the same arrangement across different populations (*F_ST_* values within arrangements). Statistical significance was tested after 10000 permutations.

#### Linkage disequilibrium between microsatellites and inversions

For each chromosome linkage disequilibrium (LD) between microsatellites and inversions was quantified with the multiallelic version of Lewontin’s *D′*-statistic, *D′*m = Σ_ij_ *p*_i_ *q*_i_|*D′*_ij_| [41] using the software PowerMarker version 3.25 [42]. Statistical significance was evaluated using the Fisher’s exact test, with *P-*values obtained after 10000 permutations and adjusted for multiple comparisons using the False Discovery Rate correction (FDR; as in [43]). The specific LD patterns between microsatellite alleles and chromosomal arrangements were assessed with an interallelic disequilibrium measure (*D′* statistic) between multiallelic markers implemented in MIDAS [44]. The significance was tested using a Chi square (χ^2^) with Yates correction. To avoid spurious patterns associations between microsatellite alleles and inversions were only considered as significant for cases with three or more observations.

#### Latitudinal microsatellite variation within arrangements

In order to search for an association of microsatellite variation with geographical distance, for each arrangement separately, linear regressions between pairwise population *F_ST_* values and the logarithm of the geographical distances (in Kms) between populations were carried out using data from each microsatellite locus independently and all loci combined. Statistical significance of these linear regressions was obtained through 10000 permutations using the SPAGeDi software v1.3 [45].

The two most common alleles for each locus, defined after averaging frequencies across populations, were analysed in order to detect specific alleles presenting clinal frequency variation. Linear regressions between the frequencies of the two most common alleles (arcsin transformed) for each locus and latitude were carried out. Regression analyses were computed with the STATISTICA 8.0 software package and FDR correction was applied to their significance levels.

A test to detect selection on microsatellite loci was carried out to identify outlier loci through the comparison of observed *F_ST_* values with a neutral distribution of expected *F_ST_* values, conditioned on heterozygosity, obtained from coalescent simulations [46]. The aim is to test for loci presenting significantly higher (positive selection) or lower genetic differentiation (balancing selection) across populations, relative to that expected under the neutral distribution. This test was applied to the microsatellite data of each chromosomal arrangement independently (comparing across populations) using the LOSITAN software ([47], available at http://popgen.eu/soft/lositan/) to generate a neutral distribution based on an island model of population structure, assuming an Infinite Allele mutation model, with 100 000 paired values of *F_ST_* and heterozygosity.

## Results

### Latitudinal Variation in Chromosomal Inversions

To confirm the maintenance of the latitudinal clines in inversion frequencies linear regressions of the arcsin transformed frequency of each arrangement with latitude were carried out. A clear linear association with latitude was found for all frequent chromosomal inversions with the exception of A_1_. A_2_, J_1_, U_1+2_and U_1+2+8_ arrangements decreased in frequency with increasing latitude while all standard arrangements increased in frequency (see Tables S1 and S2). These linear associations between inversion frequencies and latitude presented the same sign as previously reported (e.g. [5]–[7]).

ANOVAs were performed to search for differences in the variability of chromosomal arrangement frequencies across populations. No significant differences across populations were obtained in the heterozygosity for chromosomal arrangements, after arcsin transformation (F = 2.29, *P*<0.07). Nevertheless, the populations in lower and higher latitudes (respectively Iberian and Scandinavian populations) presented consistently lower H_e_ values particularly in the A and J chromosomes (see Table S1 and Figure S1). For these two chromosomes heterozygosity fit a quadratic regression with latitude (A: R^2^ = 0.74, *P* = 0.015; J: R^2^ = 0.72, *P* = 0.021). This is in accordance with the inverse latitudinal clinal frequency patterns of most inversions as previously mentioned, which results in populations from intermediate latitude presenting higher variability in chromosomal arrangements. However, for the U chromosome its inversion polymorphism did not follow the same trend due to the frequency of the U_1+2_arrangement which itself was better fit to a quadratic regression with latitude (R^2^ = 0.81, *P* = 0.007).

### Genetic Variability within and among Chromosomal Arrangements

The genetic variability in microsatellite loci was analysed in *ca*. 600 individuals: 602 individuals were genotyped for the U chromosome, 584 for the J chromosome and 592 for the A chromosome. Table 1 shows the overall levels of microsatellite genetic variability (expected heterozygosity) in microsatellite loci obtained for individuals carrying the most frequent arrangements of the A, J and U chromosomes in the 9 studied populations (see also Table S3).

**Figure 3:**
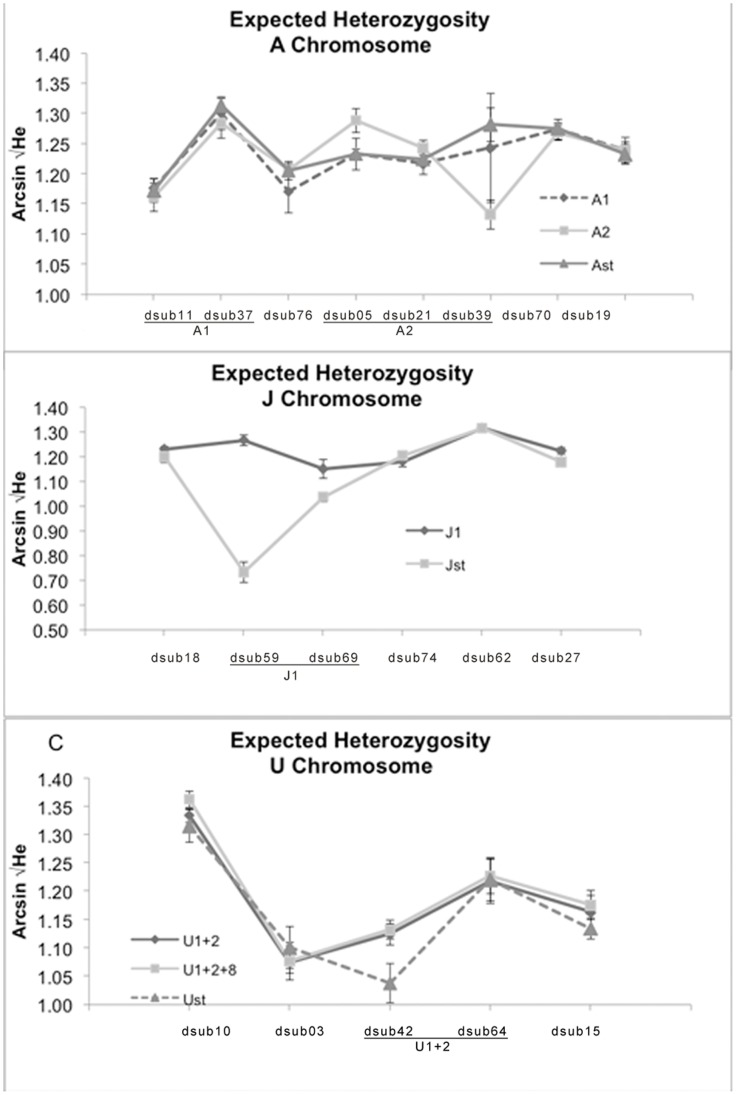
Mean Expected Heterozygosity of each microsatellite locus for the different chromosomal arrangements. Microsatellite loci are ordered by cytological location relative to the standard arrangements. A) Chromosome A, B) Chromosome J, C) Chromosome U. Bars correspond to standard errors from the different localities within arrangements.

Trifactorial ANOVAs were performed per chromosome to search for differences in genetic variability between populations, arrangements, loci and interactions between the different factors (Table S4). For the A chromosome, a trifactorial ANOVA indicated a significant effect of the interaction between population*locus and arrangement*locus. The highly significant interaction arrangement*locus is due to the low genetic variability of locus dsub39 (located within A_2_) in the individuals carrying the A_2_ inversion – see Figure 3A. A post-hoc Tukey test on this interaction term, revealed that locus dsub39 was the one presenting the highest number of significant differences both relative to other loci (in any of the arrangements being considered) and also in the same locus across arrangements (data not shown).

For the J chromosome the same trifactorial ANOVA model (Table S4) showed a significant effect of population (with the Scandinavian populations presenting consistently lower variability – Table 1), and also of the arrangement*locus interaction term. A post-hoc Tukey test indicated that for J_ST_ individuals both loci located inside the inverted region – dsub69 and mostly dsub59– presented significantly lower genetic variability than other loci of the standard chromosome and also than all loci in J_1_ individuals (Figure 3B). For the U chromosome significant differences were only found between loci (see Figure 3C). No significant locus*arrangement interaction was observed despite the fact that dsub42 presents a decrease in variability for individuals with the U_ST_ arrangement (Figure 3C).

It is important to point out that the population*arrangement interactions were always non-significant for all chromosomes, indicating no relevant differences in the pattern of variation of expected heterozygosity associated with different gene arrangements across the nine populations.

Bifactorial ANOVAs were applied specifically on the data of each arrangement, defining population as fixed factor and locus as random factor. This allowed testing directly the differences in expected heterozygosity for a particular gene arrangement across populations. This analysis showed significant differences in expected heterozygosity across populations for arrangements J_ST_ and for U_1+2_(F = 2.43, *P*<0.036 and F = 3.31, *P*<0.011, respectively). Interestingly, these differences disappear if the Scandinavian populations are excluded from the analysis, as these are the ones presenting consistently lower values of genetic diversity (Tables 1 and S3).

Patterns of molecular heterozygosity for specific loci might be affected by the frequency of particular inversions in the different populations, particularly those loci that are located within inverted regions. Nevertheless, correlations between locus heterozygosity and inversion frequencies showed that the genetic variability of a given locus was generally independent of the frequency of the inversion in a population, as correlations were not significant in 90% of the comparisons (data not shown).

### Patterns of Genetic Differentiation within and among Chromosomal Arrangements

**Figure 4:**
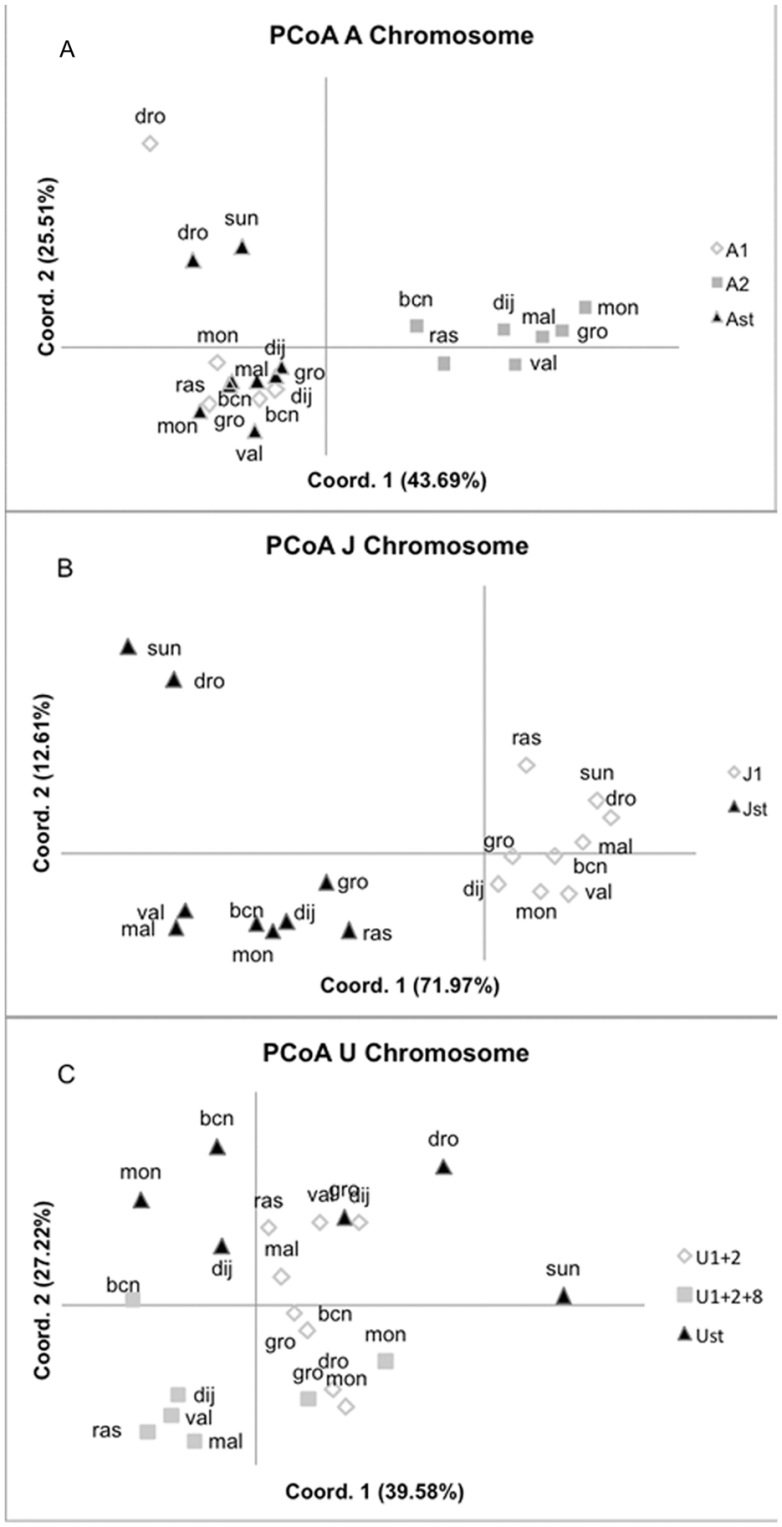
Principal Coordinate Analysis (PCoA) based on pairwise *F_ST_* values for all loci combined from each chromosomal arrangement and population. A) Chromosome A, B) Chromosome J, C) Chromosome U.

To study the genetic differentiation within and among chromosomal arrangements across populations, individuals were grouped according to the population and chromosomal arrangement they carried (e.g. individuals from Málaga carrying A_2_ inversion constituted one single analysis group; see Material and Methods). For each chromosome, between arrangements *F_ST_* analyses indicated a much higher genetic differentiation than comparisons between individuals carrying the same arrangement across populations (within-arrangement differentiation). These differences are statistically significant in chromosomes A and U: A chromosome – *F_ST_* between arrangements = 0.015, *F_ST_* within arrangements = 0.007 (Wilcoxon *P*<0.02); J Chromosome – *F_ST_* between = 0.037, *F_ST_* within = 0.007 (Wilcoxon *P*<0.08); U Chromosome – *F_ST_* between = 0.017, *F_ST_* within = 0.007 (Wilcoxon *P*<0.05). Between arrangement *F_ST_* values were statistically significant in all chromosomes (*P*<0.001), while the within arrangement *F_ST_* values were statistically significant for A_1_ (*P*<0.01) and A_ST_ (*P*<0.001); J_ST_ (*P*<0.001); U_1+2_ (*P*<0.01) and U_ST_ (*P*<0.001) – see also Figure S2 *F_ST_* values of each locus. Importantly, when excluding the Scandinavian populations from the analysis due to their consistently higher genetic differentiation relative to the other populations (see Figure 4), the within–arrangement differentiation values across populations were even lower and not significant for any arrangement. This shows that the significant within arrangement differentiation reported above for some arrangements was solely due to the effect of the Scandinavian populations. The only exceptions to this pattern were the significant differentiation in locus dsub11 for A_ST_ individuals and locus dsub03 for U_1+2_ individuals (see Figure S2). On the other hand, between arrangement differentiation remained significant in all three chromosomes (*P*<0.001). The general trend of higher between than within-arrangement differentiation was also observed when excluding the Scandinavian populations although only statistically significant in the A chromosome (A chromosome – *F_ST_* between = 0.010, *F_ST_* within = 0.0005, Wilcoxon *P*<0.02; J Chromosome – *F_ST_* between = 0.025, *F_ST_* within = 0.0001, Wilcoxon *P*<0.7; U Chromosome – *F_ST_* between = 0.008, *F_ST_* within = 0.0002, Wilcoxon *P*<0.08). Heterogeneity across loci in *F_ST_* values for the between arrangement comparisons may be responsible for the large *P*-value in the J chromosome; in fact, loci located inside inverted regions present much higher *F_ST_* values than loci located outside (*F_ST_* inside = 0.013 *vs. F_ST_* outside = 0.002 in A chromosome; *F_ST_* inside 0.064 *vs. F_ST_* outside = 0.0005 in J chromosome; *F_ST_* inside = 0.010 *vs. F_ST_* outside = 0.006 in U chromosome, see Figure S2). As such, high genetic differentiation between arrangements is found in all chromosomes mostly due to loci located inside inversions. This is particularly due to the impact of locus dsub39 (located within A_2_), dsub59 and dsub69 (located within J_1_) and dsub42 (located within U_1+2_ and U_1+2+8_), which present high values of between arrangement differentiation – see Figure S2.

For each chromosome, principal Coordinate Analyses (PCoAs) based on *F_ST_* pairwise matrices, measuring genetic differentiation within and among arrangements were computed for all loci combined (Figure 4) as well as for loci within each region independently (Figure S3).

Patterns of genetic differentiation for the A chromosome were studied for all loci combined (Figure 4A) and also separately for loci in region I that includes the A_1_ inversion, region II that includes the A_2_ inversion and region III with no inversion included (see Figure S3). Groups bearing A_2_ from different populations clustered together and were separated from the other two arrangements (A_1_ and A_ST_) by the first axis explaining 44% of the variation, while the second axis (26% of variation) separated the individuals from Scandinavian populations with inversions A_1_ and A_ST_ (Figure 4A). This pattern was mainly due to the loci in region II (inversion A_2_), with the two first axis explaining 77% of the variation while the loci on regions I and III explained lower percentages of variation (56% and 57%, respectively) and did not show such a clear clustering of individuals (see Figure S3). When *F_ST_* values were obtained for each locus independently, locus dsub39, located within A_2_, was the one explaining the highest amount of variation (two first axis explaining 80%, data not shown). Locus dsub39 not only presented the highest global *F_ST_* values, showing large differences between inversions across populations, but also significant differentiation within inversions A_1_ and A_ST_ across populations (Figure S2).

For the J chromosome, the PCoA analysis performed including all loci indicated a clear differentiation between individuals with J_ST_ and J_1_ inversions (first axis explaining 72% of the variation), with the J_ST_ inversions from the Scandinavian populations further differentiated from all others (second axis explaining 13%) (Figure 4B). This pattern was due to the loci located within the J_1_ inversion with the two first axes explaining 92% of the total variation. PCoA analysis only considering loci outside inversions separated the individuals from Scandinavian populations from all others (see Figure S3). PCoA analyses for each locus indicate that both loci located within inversion J_1_ explain the highest percentages of the amount of variation in the first two axis, with locus dsub59 and dsub69 explaining 98% and 83% of the total variation, respectively. Both these loci indicate high levels of genetic differentiation between inversions in a global *F_ST_* analysis performed across populations, while locus dsub69 also presented low but significant within-inversion differentiation across populations (Figure S2).

For the U chromosome individuals from Scandinavian populations clustered apart from the rest based on the first axis (40%) of the PCoA analysis using all loci (Figure 4C). The second axis (22%) separated mostly individuals carrying the U_1+2+8_arrangement from those carrying the other two arrangements. When only the loci located inside U_1+2_(locus dsub42 and dsub64) were used the same pattern revealed with all loci combined was observed. However, when only the loci located outside the arrangements were used, the groups were more intermixed (see Figure S3). When each locus was analysed independently, we observed that genetic differentiation was mostly due to locus dsub42– which clearly differentiates individuals carrying the three U arrangements (first two axes explained 71% of total variation, data not shown). Locus dsub42 also presented the highest and significant *F_ST_* values when comparing different U arrangements (Figure S2).

### Association Patterns between Microsatellite Loci and Inversions

The D′m statistic - multiallelic version of Lewontin’s D′ [41] was applied in order to detect non-random associations between microsatellite loci and the arrangements of each of the three chromosomes studied. Significant D′m values were mostly obtained for loci located inside inversions in the two autosomes and in region II of the sex chromosome (Table 2). However, in different populations, despite the same loci presented indications of LD, for most of them different alleles were showing significant LD with each chromosomal arrangement (data not shown). Nonetheless in three loci the same allele was in LD with the same inversion in multiple localities. For locus dsub39, located near the distal breakpoint of the A_2_ inversion (Figure 2), allele 277 (in bp) presented significant LD with the A_2_ inversion in five populations (València, Barcelona, Montpellier, Dijon and Groningen). For locus dsub59, in the J chromosome allele 245 bp was in disequilibrium with J_ST_ in all populations. Similarly, for locus dsub69 the majority of the significant LD patterns obtained with the J_ST_ inversion involved the same allele (allele 143 bp).

**Table 2:** *D′*m statistic between microsatellite loci and arrangements for each chromosome in the nine analyzed populations.

Chromosome	Locus	MAL	VAL	RAS	BCN	MON	DIJ	GRO	DRO	SUN	
**A**	dsub11	0.425	0.252	0.286	0.210	**0.397** **	0.302	0.373	**0.578** ***	0.582	
	dsub3 7	0.418	0.575	0.443	**0.588** **	0.517	0.460	0.467	0.428	0.975	
	dsub76*	**0.599** *	0.461	**0.631** ***	0.417	0.456	0.348	0.316	0.438	0.716	
	dsub05*	0.467	0.500	**0.545** **	0.470	0.455	**0.479** **	0.432	0.375	0.651	
	dsub21*	0.441	0.595	0.376	0.324	0.477	0.370	0.412	0.292	0.508	
	dsub39*	**0.622** *	**0.661** **	0.452	**0.595** **	**0.591** ***	0.411	0.427	0.455	0.571	
	dsub70	0.498	0.460	0.450	0.422	0.486	**0.502** **	0.393	0.566	0.691	
	dsub19	0.511	0.337	0.367	0.333	0.320	0.296	0.348	0.467	0.687	
**J**	dsub18	0.526	0.372	0.421	0.395	0.390	0.333	0.433	0.335	0.356	
	dsub59*	**0.731** ***	**0.738** ***	0.560	**0.749** **	0.564	**0.567** **	**0.633** ***	**0.713** ***	**0.631** ***	
	dsub69*	**0.647** ***	**0.612** ***	0.456	**0.568** ***	**0.607** **	**0.611** *	**0.500** *	**0.901** ***	**0.787** ***	
	dsub74	0.356	0.362	0.437	0.384	0.256	0.300	0.267	0.413	0.515	
	dsub62	0.495	0.310	0.519	0.440	0.445	0.467	0.467	0.413	0.561	
	dsub27	0.287	0.167	0.322	0.375	0.393	0.367	0.467	0.500	–	
**U**	dsub10	0.495	0.407	0.450	0.491	0.596	0.581	0.573	0.418	0.470	
	dsub03	0.329	0.248	0.450	0.267	0.336	**0.324** *	0.239	0.486	0.450	
	dsub42*	**0.430** *	0.406	**0.467** *	**0.381** *	0.280	**0.392** ***	**0.380** *	**0.357** **	0.774	
	dsub64*	0.369	0.369	0.333	0.460	**0.604** **	0.379	0.477	0.463	0.683	
	dsub15	0.260	0.407	0.400	0.423	0.284	**0.424** **	0.247	0.361	0.309	

Note. Loci inside region II (including A_2_ inversion) and inside inversions in the J and U chromosomes are indicated with an asterisk. (−) Data not amplified. Significant *P*-values are highlighted in bold;

*** *P*<0.001;

** *P*<0.01;

* *P*<0.05.

In order to detect regions within chromosomes presenting possible epistatic interactions, non-random associations between loci within the chromosomes carrying the same inversion were assessed. For chromosomes carrying the A_ST_ inversion, non-random associations were detected between dsub19 and dsub39 and between dsub5 and dsub37 for both Sunne and Drøbak (Fisher’s Exact test, *P*<0.05). Associations were also found between dsub19 and dsub37 and also between dsub5 and dsub19 for the Rasquera and Sunne populations (Fisher’s Exact test, *P*<0.05). In general, no consistent associations across populations were found between loci in the other inversions of the sex chromosome or in the autosomes. To increase the sample size and thus also the statistical power to detect LD patterns, all individuals from different populations carrying the same inversion were grouped. In these analyses the Scandinavian populations were excluded, as they were the ones generating within-arrangement differentiation across populations (see *F_ST_* analyses reported above). In the U chromosome LD was detected between loci dsub15 and dsub42 (Fisher’s Exact test, *P*<0.05) for the individuals carrying the U_1+2+8_ arrangement. Interestingly, multi-locus LD in A_ST_ chromosomes was also detected for loci dsub39-dsub70-dsub19, located in the distal part of the A chromosome (Fisher’s Exact test, *P*<0.05).

Hierarquical analysis of Linkage Disequilibrium [48] using Linkdos [49] also did not suggest epistatic selection between loci of chromosomes carrying the same inversion as, in all chromosomes, variance of LD among populations D´^2^_IS_ was always substantially higher than variance of LD within the overall population D´^2^_ST_ (data not shown). This result points to different LD patterns between loci across the different populations, probably due to genetic drift or other stochastic events as selection would most likely generate similar LD patterns across populations [48].

### Geographical Clinal Patterns of Genetic Variation

Clinal variation in the genetic content of each chromosomal arrangement was analysed at a broad geographical scale ranging about 3700 km by comparing the genetic distance between populations and their geographic distance. Linear regressions of pairwise *F_ST_* values between populations on the logarithm of geographical distances were calculated using information from all loci combined and each locus separately. For the A_1_ inversion only locus dsub5 presented a significant association with distance (R^2^ = 0.55; *P* = 0.008, 10000 permutations of individuals and populations) after FDR correction. On the other hand, in the A_ST_ inversion significant associations of *F_ST_* values with geographical distance were found for locus dsub39 and for all loci combined (R^2^ = 0.40, *P* = 0.007 and R^2^ = 0.37, *P* = 0.016, respectively). For the J_ST_ inversion all loci combined (R^2^ = 0.43; *P* = 0.008) as well as several loci alone dsub18 (R^2^ = 0.47; *P* = 0.004), dsub69 (R^2^ = 0.25; *P* = 0.007) and dsub62 (R^2^ = 0.36; *P* = 0.007) presented significant association between genetic differentiation and geographical distance. For the U chromosome, there was only a significant association between genetic and geographic distances for the U_ST_ inversion when information from all loci was combined (R^2^ = 0.29; *P* = 0.007).

The frequencies of the most common alleles of each locus were plotted against latitude to pinpoint specific microsatellite alleles presenting clinal variation. Table 3 shows the associations of the most common (MCA) and second most common (SMCA) microsatellite alleles with latitude (see allele frequencies of all loci across arrangements and populations in Table S5). The only significant clinal patterns after FDR were found for locus dsub39 in individuals with the A_2_ inversion (SMCA, 278 bp; R^2^ = 0.870), and for locus dsub42 in individuals with U_1+2_arrangements (MCA, 126 bp; R^2^ = 0.780) – Figure S4. Noticeably, these alleles belong to loci located within inverted regions presenting significant patterns of linkage disequilibrium with inversions (Table 2).

**Table 3:** Regression coefficients of the frequencies of the most common allele (R^2^_mca_) and second most common allele (R^2^_smca_) with latitude in individuals carrying the same chromosome arrangement.

	R^2^_mca_			R^2^_smca_		
Locus	A_ST_	A_1_	A_2_	A_ST_	A_1_	A_2_
dsub11	0.001	0.647	0.030	0.226	0.681	0.171
dsub37	0.006	0.307	0.170	0.113	0.085	0.010
dsub76*	0.066	0.550	0.514	0.374	0.722	0.117
dsub05*	0.093	0.321	0.693	0.407	0.886	0.108
dsub21*	0.241	0.000	0.025	0.027	0.005	0.192
dsub39*	0.340	0.563	0.091	0.409	0.563	**0.870**
dsub70	0.426	0.018	0.170	0.017	0.066	0.112
dsub19	0.574	0.436	0.000	0.314	0.747	0.074
Locus	J_ST_	J_1_		J_ST_	J_1_	
dsub18	0.000	0.117		0.236	0.000	
dsub59*	0.006	0.005		0.018	0.303	
dsub69*	0.029	0.372		0.369	0.058	
dsub74	0.005	0.293		0.126	0.073	
dsub62	0.403	0.274		0.235	0.433	
Locus	U_ST_	U_1+2_	U_1+2+8_	U_ST_	U_1+2_	U_1+2+8_
dsub10	0.115	0.582	0.603	0.176	0.200	0.475
dsub03	0.346	0.583	0.194	0.132	0.176	0.016
dsub42*	0.524	**0.780**	0.033	0.347	0.472	0.033
dsub64*	0.311	0.337	0.124	0.074	0.298	0.367
dsub15	0.570	0.006	0.658	0.729	0.457	0.006

Note. Loci inside region II (including A_2_ inversion) and inside inversions in the J and U chromosomes are indicated with an asterisk. Significant *P*-values after False Discovery Rate (*P*<0.05) are highlighted in bold.

In order to detect a signature of selection within inversions, we used a coalescent simulation model implemented in LOSITAN [47] to detect outlier loci with high *F_ST_* values, given their observed heterozygosity, as an indication of positive selection. Locus dsub39 was the only locus identified as being an outlier in individuals carrying the A_1_ and the A_ST_ inversions (*P*<0.001, in both cases).

## Discussion

This study is the widest geographical analysis performed on the molecular variation associated with clinal inversion polymorphism in *Drosophila subobscura* and clearly shows the impact of chromosomal arrangements in shaping the molecular genetic patterns of the European populations of this species. We found large genetic differentiation between individuals carrying different arrangements and showed this was caused by loci mapping inside arrangements. Importantly, these patterns were consistent across chromosomes and populations at a wide geographical scale. The most likely explanation for our results is the reduction of recombination between chromosomal arrangements, with loci within and near breakpoints of inverted regions presenting restricted gene flux [19],[50],[51] with higher gene exchange expected to occur towards the middle of inversions [52], [53]. Particularly relevant in our study is the fact that, even within the same population, significant overall genetic differentiation was obtained between individuals carrying different arrangements presumably due to this specific effect of reduced recombination in heterokaryotypes [50]. This is relevant as it highlights the importance of taking into account prior information on inversion polymorphisms when analysing broad molecular genetic patterns particularly in species such as *D. subobscura*, which present a high number of inversions in the genome.

The patterns of Linkage Disequilibrium reported in our study also support an important role of gene arrangements in shaping the molecular genetic content of the chromosome. The stronger LD levels were obtained for loci located inside arrangements (see also [14], [21], [54], [55]). The most striking examples were locus dsub39 located within the A_2_ inversion (near its distal breakpoint) and dsub59 located inside J_1_ (in the middle of the inversion). In these loci, the same allele is in LD with the inversion in different populations indicating either a selective effect, association due to a bottleneck event or the origin of the inversion. In addition, variability patterns in these loci were also clearly influenced by their location inside inverted regions. The region within J_ST_ nearby the dsub59 locus might in fact mark a region subjected to selection (see below).

### Similarity of Genetic Content within Arrangements Across Populations

A clear finding of this study is the overall low levels of genetic differentiation in a given gene arrangement across a large number of European populations distributed along a latitudinal cline covering >3000 Km. Similarly, no geographic differentiation, based on nucleotide polymorphism, was observed within chromosomal arrangements of the O chromosome (Muller’s E element) in two south European populations of *D. subobscura* [22]. Also, no genetic differentiation was observed within arrangements of Muller’s C element of *D. pseudoobscura* across four American populations [14]. Importantly, our observed pattern of very low genetic differentiation was consistent across loci and arrangements in all chromosomes analysed, which reinforces the validity of this finding.

The low genetic differentiation within arrangements across populations is most likely due to the occurrence of important gene flow in this species [11], [56] coupled with the free recombination in homokaryotypes and recombination reduction in heterokaryotypes [50]. Differences in the genetic content of particular chromosomal arrangements across populations were only found in comparisons including the Scandinavian populations (see Figures 4, S2 and S3). It is unlikely that these differences arose from sampling across years as other studies have shown general stability of allele frequencies in *Drosophila* populations collected in different years [57], [58]. Furthermore, another study [11] reported lower genetic variability and higher genetic differentiation in a population of *D. subobscura* from northern Europe relative to more central European populations. Thus, the genetic differentiation detected in the present work relative to Scandinavian populations is more likely associated with restriction to gene flow due to the geographic barrier constituted by the North Sea. Also, lower effective size of the Scandinavian populations might be a factor, supported by the overall lower genetic variability of these northern populations.

These findings do not agree with the expectation of the coadaptation model of differences in the genetic content of a given gene arrangement across populations as a result of contrasting selective scenarios in different environments [12]. Moreover, the absence of clear LD patterns between microsatellite loci located within arrangements in a given chromosome does not point to the existence of epistatic interactions within arrangements, which is also central to the coadaptation model. In this context, the reported extensive LD between loci located in the distal part of the A chromosome in A_ST_ individuals could be due to low recombination in the region concerned or physical constraints and not to any selective process. Evidence for epistasis interactions was reported in a study of 8 gene regions in *D. pseudoobscura* based on linkage disequilibrium among them [14]. LD patterns indicative of epistasis were also found in molecular markers located within and near In(3R)Payne in *Drosophila melanogaster* [21]. Several reasons may explain the inability to detect clear indications of epistatic interactions and also the overall absence of genetic differentiation within arrangements across populations in our study. For instance, it is possible that the maintenance and evolution of inversions does not necessarily involve epistatic effects but merely result from the cumulative positive effects of genes involved in local adaptation such as hypothesized by Kirkpatrick and Barton [15]. These inversions may then spread due to migration, without expecting major changes in their genetic content as observed in our study.

Alternatively, if few genes within inversions are involved in epistatic selection and the regions covered by our microsatellite loci are not located near selected regions we might be unable to detect associations among loci. Studies involving a higher number of markers, with a wider coverage of the inversions will allow higher resolution power to test if this indeed was the case and also if low within-arrangement genetic differentiation across populations is in fact the rule. Massive parallel sequencing methods, and the approaching possibility to obtain the entire genome sequence of *Drosophila subobscura* will be crucial in this regard.

Nonetheless different processes might affect different inversions, chromosomes and even species. In this context, the reported extensive LD between loci located in the distal part of the A chromosome in A_ST_ individuals could be indicative of epistatic interactions and selection, although it might also be explained by low recombination in the region.

### Is there Evidence of Selection in Some Inversions?

Individuals carrying the J_ST_ presented significantly lower variability than those carrying the J_1_ inversion. This could indicate a selective sweep event in the region nearby locus dsub59 thus leading to a decrease in variability, although other possible alternative explanations exist, such as (1) a recent bottleneck associated with the J_ST_ inversion or (2) an historical effect due to the possible recent origin of this inversion. Nevertheless these alternative explanations appear less likely since in (1) a bottleneck would most likely produce a reduction in variability in all loci within the inversion and not just around a particular locus; and in (2) an historical effect would be most susceptible to be observed in a derived inversion while J_ST_ is thought to be the ancestral arrangement, based on phylogenetic studies of inversions in the *obscura* group [3]. A third possible non-selective explanation might refer to the lack of double cross-overs within the inverted region and hence low levels of recombination with consequent low genetic variation in this region. This could be a relevant point specifically given the relative low length of the inverted region considered - approximately 22 cM [55] – which would render negligible the effect of double cross-overs within the inversion [19], [59]. However, other loci located inside/near the breakpoint of the same inversion (e.g., dsub69 and dsub74) did not show such reduced variability. Furthermore, under extremely low recombination levels we would expect LD also occurring between loci within the considered inversion, which is not the case. All in all, this points to a possible region of low variability associated with selective causes although further analyses specifically focusing on this particular locus and the nearby region within the J_ST_ inversion are needed.

### Is Selection Promoting Clinal Variation of Alleles within Inversions?

Despite the low genetic differentiation within arrangements across populations, there were some indications of within-arrangement variation in allele frequencies against latitude in our study. As discussed above, this was mainly due to the Scandinavian populations, as inversions from these populations were the ones presenting higher differentiation in their genetic content relative to others. This might also explain the fact that the overall patterns of clinal variation were higher in the “standard” chromosomes since these present higher frequencies in the Scandinavian populations. This overall pattern is most likely due to demographic factors, since this sign of isolation by distance is seen at several loci of different chromosomes, and particularly when information from all loci is taken together.

Interestingly, we also found specific alleles presenting changes in frequencies highly correlated with latitude which are not explained by the effect of Scandinavian populations. It is revealing that those presenting the stronger sign of clinal variation correspond to alleles of microsatellite loci located within inversions. This is the case of locus dsub39 within the A_2_ inversion and dsub42 located inside the U_1+2_arrangement. Coincidentally, these two loci also presented the highest levels of LD with inversions. This pattern is suggestive of clinal selection in regions surrounding the aforementioned loci. Also, locus dsub39 presents indications of higher differentiation across populations than expected under the neutral model. The fact that these clinal patterns occur in loci that map inside inverted regions suggests these gene arrangements may protect specific combinations of alleles possibly under climatic selection from the effects of recombination [1], [21], [60] and, at the same time, enhance a hitchhiking effect on alleles of microsatellite loci located nearby. In fact, the two abovementioned microsatellite loci are located in regions that are expected to have very low recombination as dsub39 is close to the breakpoint of inversion A_2_ and dsub42 maps inside a region covered by multiple inversions (with the U_1+2_, U_1+2+8_ and U_ST_ arrangements). It will thus be interesting to analyse possible candidate genes associated with thermal adaptation located in the vicinity of the microsatellite loci described above.

In summary, our data indicates a general pattern of low within arrangement differentiation across populations and no consistent indications of epistasis. As proposed in [15] we can consider as a general scenario that different inversions spread in a given population as they “harbour” different combinations of alleles with positive fitness effects in a given environment, without necessarily interacting epistatically. The clinal patterns suggestive of selection in some specific alleles within inversions indicate that the genomic content of some arrangements may vary latitudinally, eventually suggesting that different evolutionary mechanisms can be involved in the adaptive success of inversions. Furthermore, the existence of heterogeneous environments at a wider geographical scale as well as frequency-dependent selection may contribute to the maintenance of different chromosomal inversions with different fitness values in different environments thus leading to clinal variation (see also [2], [14], [61]–[63]).

Overall our study demonstrates the effective role of chromosomal inversions in maintaining different genetic pools even in the presence of high levels of gene flow along the *D. subobscura* European cline. Inversions represent an important barrier to gene flux and can thus contribute to maintain specific allelic combinations locally adapted with positive effects on fitness.

## Supporting Information

Figure S1 **Expected heterozygosity (H_e_) for chromosomal arrangements in the U, A and J chromosome plotted against latitude.**
(DOCX)

Figure S2 **Genetic differentiation (** ***F_ST_*** **) between and within chromosomal arrangements across populations.** The first column represents the global differentiation for each locus, including both between and within-arrangement differentiation across populations (All); following columns represent differentiation within each of the different arrangements of the chromosome across all populations. Asterisks represent levels of significance at: *P*<001 (***); *P*<0.01 (**); *P*<0.05 (*).
(DOCX)

Figure S3 **Principal Coordinate Analysis (PCoA) based on** ***F_ST_*** **values (see details in the Material and Methods).**
(DOCX)

Figure S4 **Microsatellite alleles presenting significant clinal variation within a given arrangement across populations.**
(DOCX)

Table S1 **Frequency of chromosomal arrangements in the nine studied populations.**
(XLSX)

Table S2 **Regression coefficients and significance of inversion frequencies plotted against latitude.**
(XLSX)

Table S3 **Levels of genetic variability (expected heterozygosity) of all loci for the populations and arrangements analysed.**
(XLSX)

Table S4 **ANOVA for each chromosome for the effects of population and arrangement as fixed factors and locus as random factor on the arcsin tranformed expected heterozygosity.**
(XLSX)

Table S5 **Microsatellite allele frequencies in individuals carrying different inversions.** A) A_ST_ inversion; B) A_1_ inversion; C) A_2_ inversion; D) J_ST_ inversion; E) J_1_ inversion; F) U_ST_ arrangement; G) U_1+2_ arrangement; H) U_1+2+8_ arrangement.
(XLS)
