# Supplementary figures and images for: The Genetic Content of Chromosomal Inversions across a Wide Latitudinal Gradient
Source: PLoS One. 2012 Dec 18;7(12):e51625. doi: 10.1371/journal.pone.0051625 (PMC3525579; doi:10.1371/journal.pone.0051625)

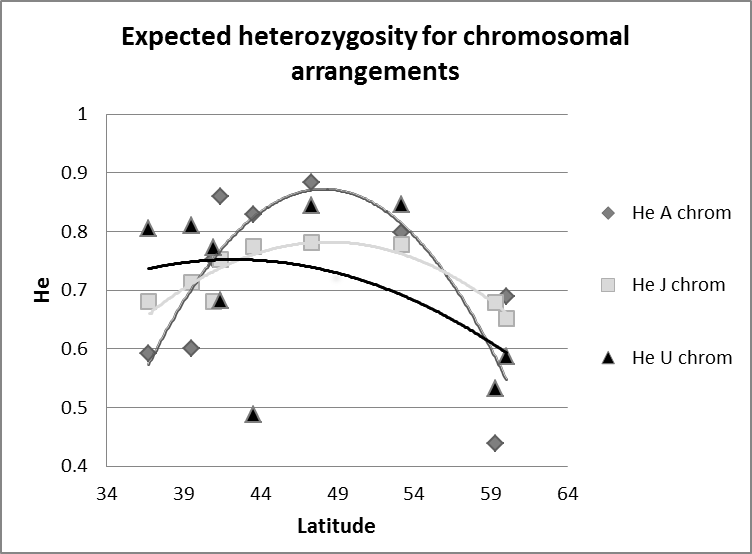

Supplement: Figure S1 — Expected heterozygosity (He) for chromosomal arrangements in the U, A and J chromosome plotted against latitude. (DOCX) [file pone.0051625.s001.docx]

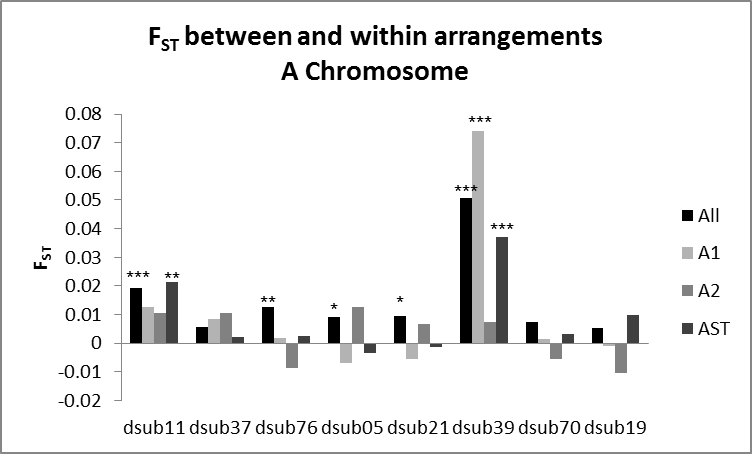


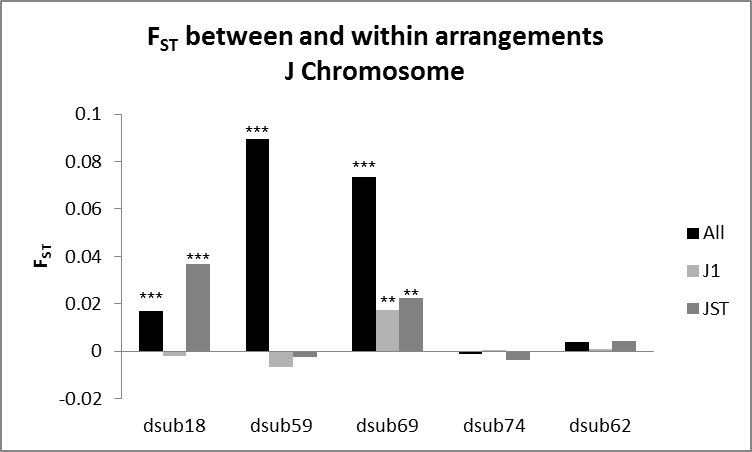


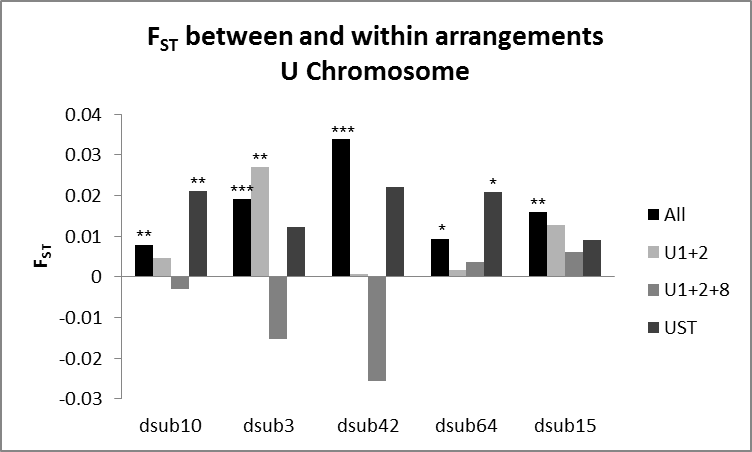


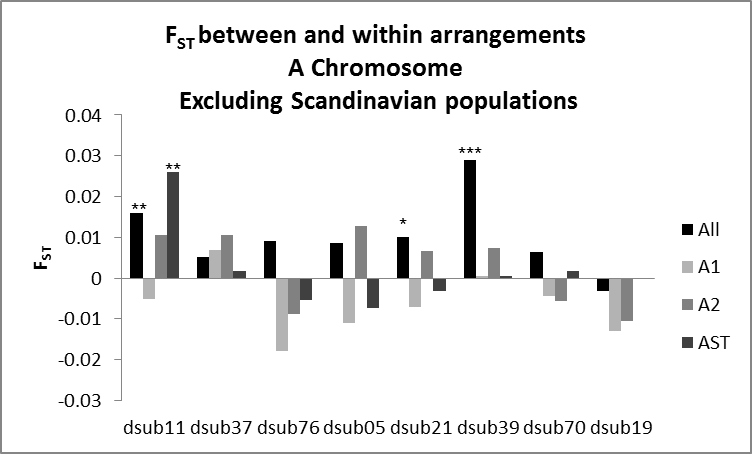


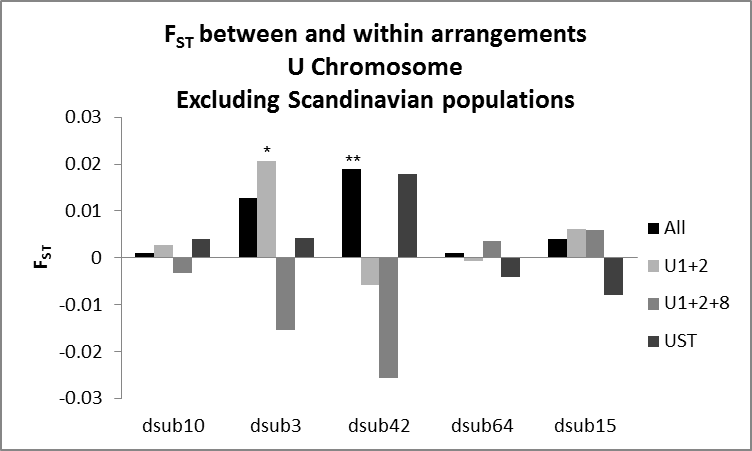

Supplement: Figure S2 — Genetic differentiation ( FST ) between and within chromosomal arrangements across populations. The first column represents the global differentiation for each locus, including both between and within-arrangement differentiation across populations (All); following columns represent differentiation within each of the different arrangements of the chromosome across all populations. Asterisks represent levels of significance at: P<001 (***); P<0.01 (**); P<0.05 (*). (DOCX) [file pone.0051625.s002.docx]

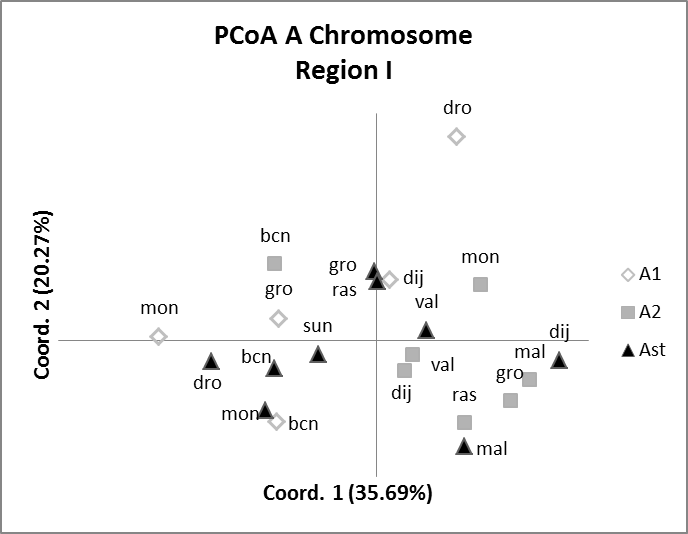


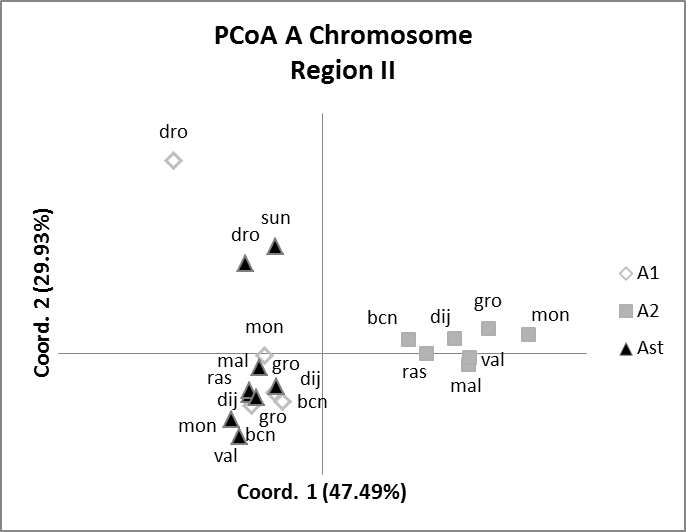


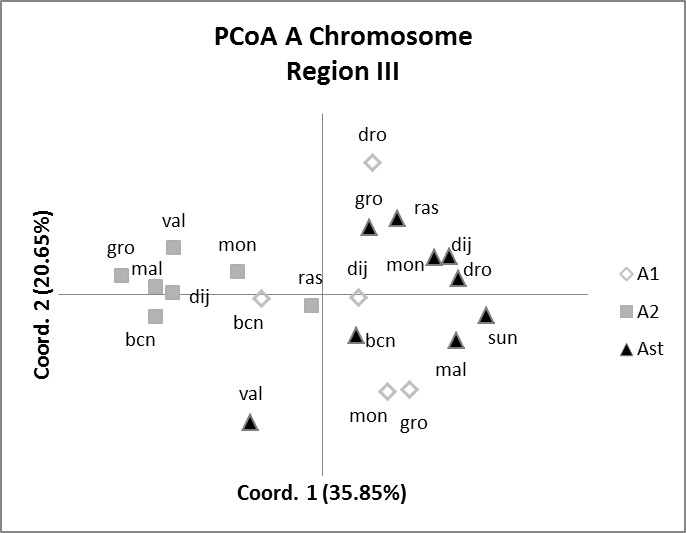


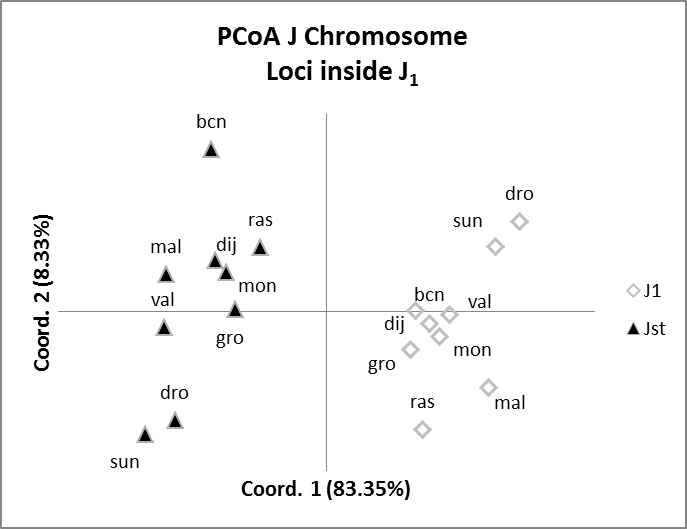


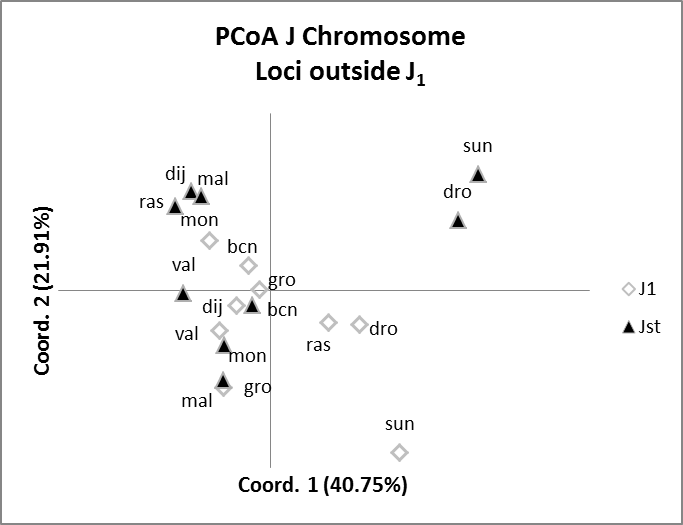


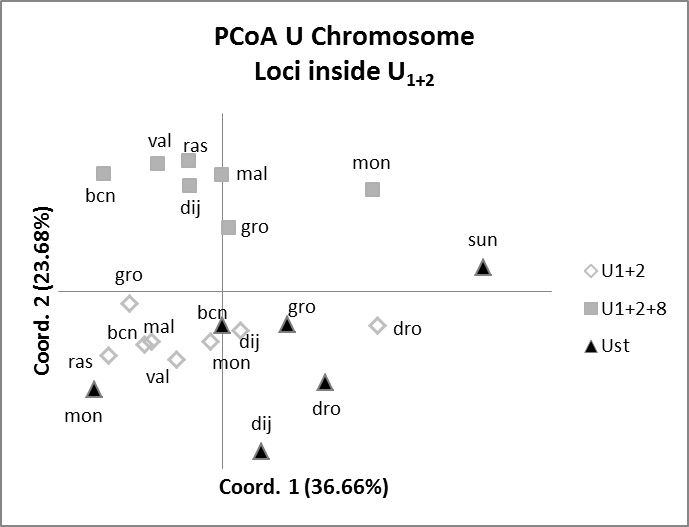


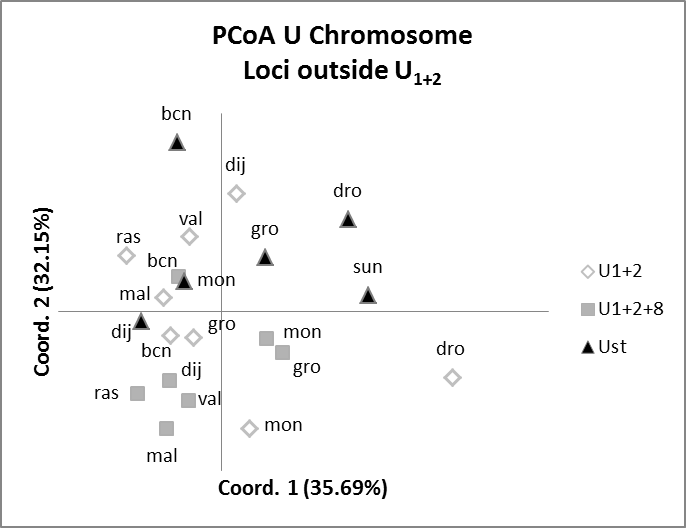

Supplement: Figure S3 — Principal Coordinate Analysis (PCoA) based on FST values (see details in the Material and Methods). (DOCX) [file pone.0051625.s003.docx]

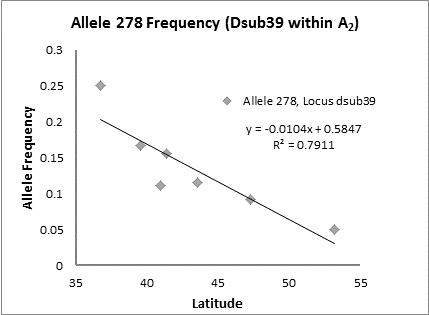


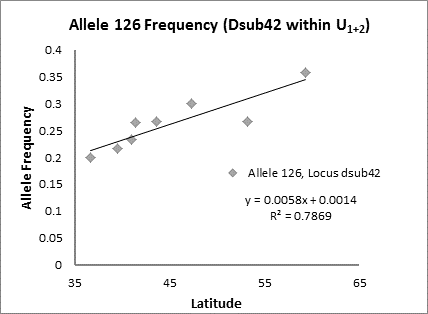

Supplement: Figure S4 — Microsatellite alleles presenting significant clinal variation within a given arrangement across populations. (DOCX) [file pone.0051625.s004.docx]
